# Comparison between partial ulnar and intercostal nerve transfers for reconstructing elbow flexion in patients with upper brachial plexus injuries

**DOI:** 10.1186/1749-7221-5-4

**Published:** 2010-01-26

**Authors:** Ryosuke Kakinoki, Ryosuke Ikeguchi, Scott FM Dunkan, Ken Nakayama, Taiichi Matsumoto, Soichi Ohta, Takashi Nakamura

**Affiliations:** 1 Department of Orthopedic Surgery, Graduate School of Medicine, Kyoto University, 54 Shougoin Kawahara-cho, Sakyo-ku, Kyoto 606-8507, Japan; 2 Department of Rehabilitation Medicine, Kyoto University Hospital 54 Shougoin Kawahara-cho, Sakyo-ku, Kyoto 606-8507, Japan; 3 Department of Orthopedic Surgery, Mayo Health System, Owatonna Clinic, 2200 26th Street, Owatonna, MN 55060, USA; 4 Department of Orthopedic Surgery, Shizuoka Prefectural General Hospital, Aoi-ku, Shizuoka, Shizuoka, Japan; 5 Department of Orthopedic Surgery, Kurashiki General Hospital, Kurashiki, Japan

## Abstract

**Background:**

There have been several reports that partial ulnar transfer (PUNT) is preferable for reconstructing elbow flexion in patients with upper brachial plexus injuries (BPIs) compared with intercostal nerve transfer (ICNT). The purpose of this study was to compare the recovery of elbow flexion between patients subjected to PUNT and patients subjected to ICNT.

**Methods:**

Sixteen patients (13 men and three women) with BPIs for whom PUNT (eight patients) or ICNT (eight patients) had been performed to restore elbow flexion function were studied. The time required in obtaining M1, M3 (Medical Research Council scale grades recovery) for elbow flexion and a full range of elbow joint movement against gravity with the wrist and fingers extended maximally and the outcomes of a manual muscle test (MMT) for elbow flexion were examined in both groups.

**Results:**

There were no significant differences between the PUNT and ICNT groups in terms of the age of patients at the time of surgery or the interval between injury and surgery. There were significantly more injured nerve roots in the ICNT group (mean 3.6) than in the PUNT group (mean 2.1) (*P *= 0.0006). The times required to obtain grades M1 and M3 in elbow flexion were significantly shorter in the PUNT group than in the ICNT group (*P *= 0.04 for M1 and *P *= 0.002 for M3). However, there was no significant difference between the two groups in the time required to obtain full flexion of the elbow joint with maximally extended fingers and wrist or in the final MMT scores for elbow flexion.

**Conclusions:**

PUNT is technically easy, not associated with significant complications, and provides rapid recovery of the elbow flexion. However, separation of elbow flexion from finger and wrist motions needed more time in the PUNT group than in the ICNT group. Although the final mean MMT score for elbow flexion in the PUNT group was greater than in the ICNT group, no statistically significant difference was found between the two groups.

## Background

In 1994, Oberlin et al. performed partial ulnar nerve transfer (PUNT) to a branch of the musculocutaneous nerve (MCN) innervating the biceps brachii muscle (BBM) on patients with upper brachial plexus injuries (BPIs) and reported successful elbow flexion function without significant neurological deficits in the ulnar nerve [1]. In their procedure, because a part of the ulnar nerve can be harvested at the level of the BBM branch of the MCN, there is a short distance needed for nerve fibers to regenerate and reinnervate the BBM. This results in the rapid restoration of function in the BBM. Because the partial ulnar nerve (PUN) was connected directly to the branch of the BBM, all of the PUN nerve fibers extended to reinnervate the BBM. Moreover, the ulnar nerve contains many motor axons, which is beneficial to motor recovery [1,2].

Intercostal nerve transfer (ICNT) was first described by Seddon, who transferred intercostal nerves (ICNs) to the MCN with sural nerve interposition in patients with BPIs [3]. Tsuyama and Hara [4] performed direct connection of the ICNs to the MCN and reported excellent outcomes for elbow flexion. ICNT can be used in patients with all types of BPI, including total brachial plexus nerve palsy. However, because the distance between the site of the neurorrhaphy and the motor point of the MCN is longer than with PUNT, it is likely that ICNT would require a longer time to reinnervate the BBM than PUNT. In addition, ICNT is associated with increased risks of pneumothorax or pneumohemothorax during surgery [4,5].

We agree that reinnervation of the BBM after PUNT is faster than that after ICNT. Patients with PUNT flex the elbow by applying forces to the muscles innervated by the ulnar nerve, such as flexor carpi ulnalis, flexor profundus muscles of the ulnar fingers, hypothenar muscles or interosseous muscles. We have often observed that, following PUNT, patients demonstrate difficulty in elbow flexion when the wrist and fingers of the affected upper extremity are extended.

In this study, we investigated patients with BPIs receiving PUNT or ICNT to restore elbow flexion and compared the outcomes. We recorded the times required to obtain Medical Research Council scale grades M1 and M3 in elbow flexion, the time required for the full range of elbow motion against gravity with maximally extended wrist and fingers, and the final outcomes of elbow flexion power.

## Methods

Among twenty BPI patients who had undergone transfer of a part of the ulnar nerve or ICNs to the MCN from 2001 to 2008, sixteen patients were enrolled in this study. Three patients (two with ICNT and one with PUNT) did not attend the postoperative rehabilitation program and were excluded from this study. Another patient with PUNT was excluded from this study because of the limited range of motion of the elbow joint of the affected upper limb after trauma that caused dislocation of the elbow joint associated with the BPI. Before the nerve transfers, exploration of the affected brachial plexuses and intraoperative somatosensory evoked action potential studies were performed on all patients to assess the brachial plexus injuries. Eight patients (six men and two women) who had avulsion injuries of C5 and C6 or C5-C7 nerve roots underwent transfer of a part of the ulnar nerve as described by Oberlin et al. [1] (PUNT group). Seven patients sustained injuries at the C5 and C6 nerve roots, and one had a C5-C7 nerve root injury that was associated with ipsilateral multiple rib fractures. The age of the patients at the time of surgery ranged from 18 to 65 years (mean 38 years). The follow-up period ranged from 51 to 403 weeks after surgery (mean 141 weeks). The mean interval between injury and surgery was 20 weeks (range 15-25 weeks; Table 1).

**Table 1:** Preoperative Data of the PUNT Group

Pt	Age/Gender	Injured NRs	Number of NRs	Injury-Surgery (W)	F/U(W)
1	65/F	C56	2	15	403

2	30/M	C56	2	16	163

3	30/F	C567	3	20	170

4	45/M	C56	2	22	99

5	28/M	C56	2	21	51

6	31/M	C56	2	25	60

7	56/M	C56	2	23	121

8	18/M	C56	2	20	58

Note. NR: nerve roots, F/U: follow up period, M: male, F: female

The other eight patients (seven men and one woman) who had C5-C7 or C5-C8 avulsion nerve root injuries underwent transfer of two ICNs to the MCNs (ICNT group). The injury levels were C5-C7 nerve roots in three patients and C5-C8 nerve roots in five patients. The age of the patients at the time of surgery ranged from 19 to 62 years (mean 38). The follow-up period ranged from 60 to 221 weeks after surgery (mean 131). The mean interval between the injury and surgery was 20 weeks (range 12-26 weeks; Table 2).

**Table 2:** Preoperative Data of the ICNT Group

Pt	Age/Gender	Injured NRs	Number of NRs	Injury-Surgery (W)	F/U(W)
9	35/M	C567	3	22	166

10	58/M	C567	3	26	152

11	19/M	C5678	4	19	132

12	56/M	C567	3	23	84

13	62/M	C5678	4	19	221

14	31/F	C5678	4	22	60

15	24/M	C5678	4	13	108

16	22/M	C5678	4	12	122

Note. NR: nerve roots, F/U: follow up period, M: male, F: female

### Surgery

**Figure 1:**
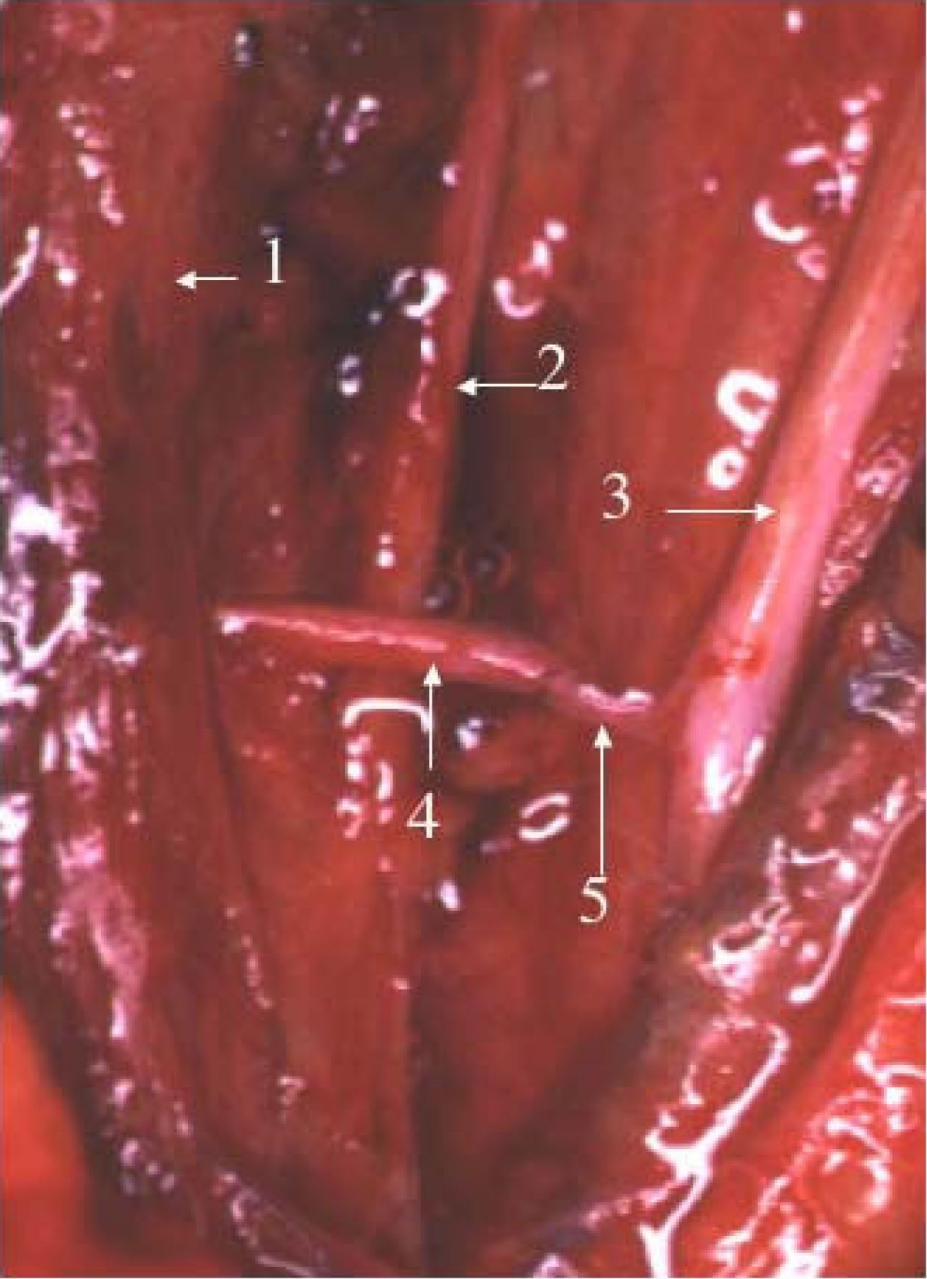
**Partial ulnar nerve transfer**. Partial ulnar nerve transfer to a branch of the musculocutaneous nerve innervating the biceps brachii muscle. 1: Musculocutaneous nerve, 2: Median nerve, 3: Ulnar nerve, 4: A branch of the musculocutaneous nerve innervating the biceps brachii muscle, 5: A part of the ulnar nerve.

In the PUNT group, the ulnar nerve and the MCN were exposed in the proximal one-third of the upper arm. Two funiculi (about 10% of the area of the transverse section of the entire ulnar nerve) were separated from the lateral surface of the ulnar nerve for 2 cm at the level where a branch innervating the BBM separated from the MCN. The ulnar nerve funiculi were divided distally and approximated to the BBM branch of the MCN (Figure 1).

**Figure 2:**
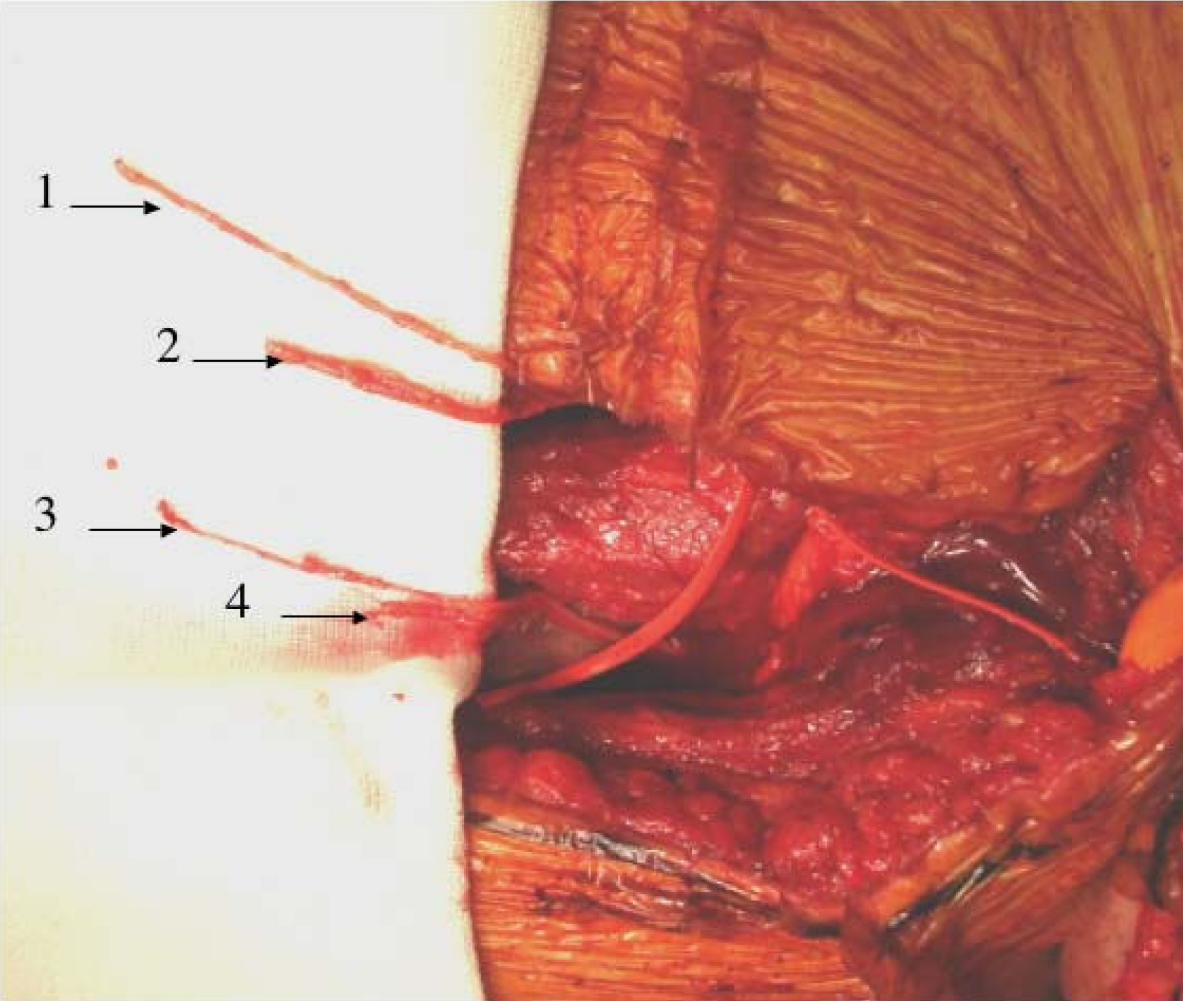
**Intercostal nerve transfer - harvesting intercostals nerves**. Exploration of the 4^th ^and 5^th ^intercostals nerves. 1, 3: The motor branches (main trunks) of the 4^th ^and 5^th ^intecostal nerves, 2, 4: The sensory branches (the lateral branches) of the 4^th ^and 5^th ^intercostal nerves.

**Figure 3:**
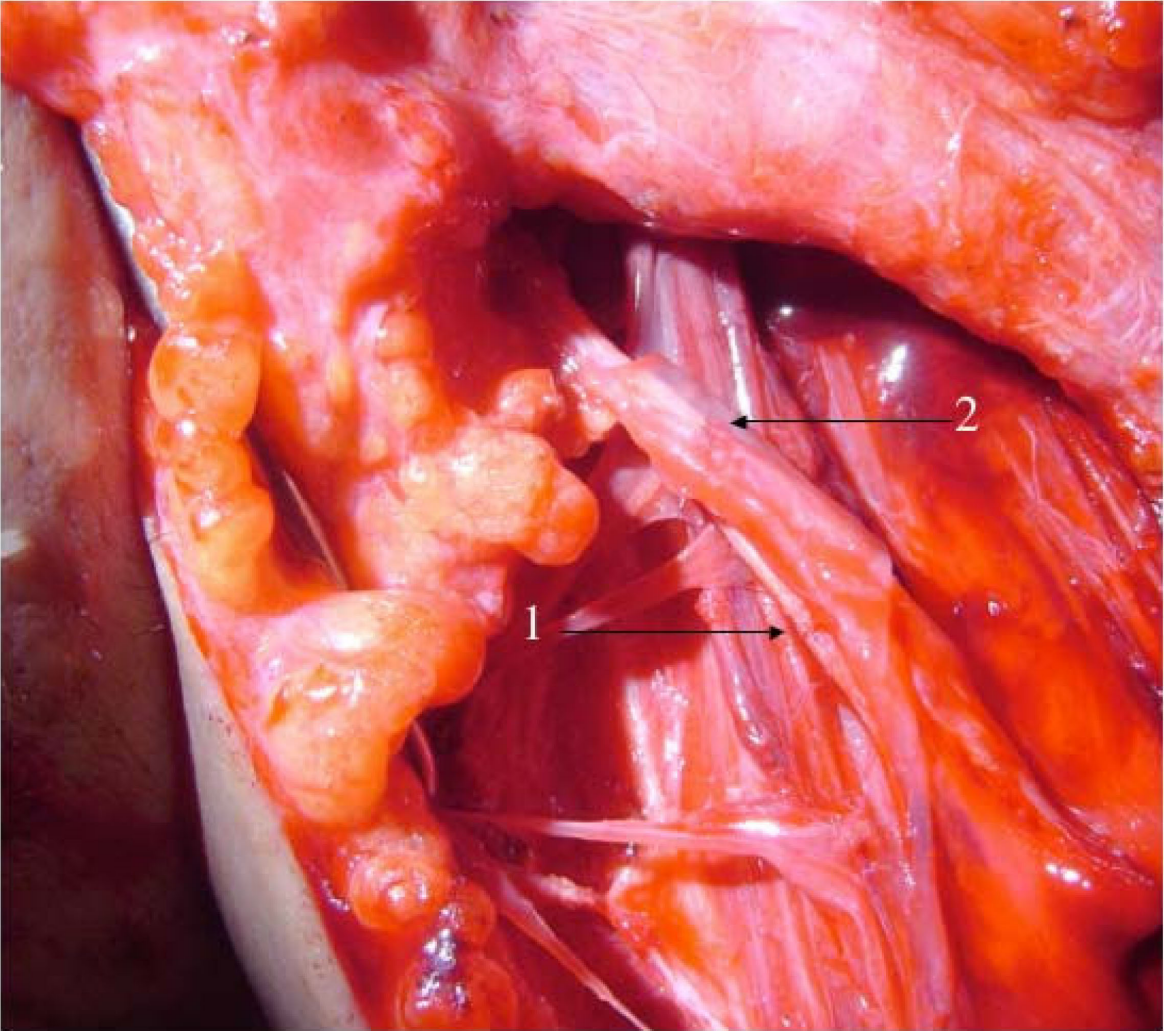
**Intercostal nerve transfer -- anastomosis between two intercostals nerves and the musculocutaneous nerve**. An intraoperative photo demonstrating intercostal nerve transfer to the musculocutaneous nerve. 1: The motor segment of the musculocutaneous nerve coaputated with two motor branches of the intercostal nerves, 2: The sensory segment of the musculocutaneous nerve approximated with two sensory branches of the intercostal nerves (fibrin glue was added at the site of the neurorrhaphy).

In the ICNT group, because the ICN bifurcates into a main trunk mainly innervating the intercostal muscles (motor branch) and a lateral branch mainly serving the sensation of the anterior chest (sensory branch) along the anterior axillary line, the ICN was elevated medially from the midaxillary line. The motor branch was sectioned at the level around the osteochondral junction of the ribs. The sensory branch of the ICN was exposed distally as far as possible (Figure 2). Elevation of both branches was performed on two ICNs (usually the 4^th ^and 5^th^, or the 5^th ^and 6^th^). The MCN was exposed in the space between the long and short heads of the BBM. Intraneural dissection was carried out proximally, and a branch innervating the biceps brachii muscle (the motor segment) was separated from a branch innervating the lateral forearm skin (the sensory segment, which contains not only sensory axons but also axons innervating the medial part of the brachial muscle; Figure 3). The two motor branches and two sensory branches of the ICN were connected to the motor segment (a branch of the MCN innervating the biceps brachii muscle) and the sensory segment (part of the MCN mainly serving for sensation of the lateral forearm skin) of the MCN, respectively (Figure 3).

All patients had additional surgery to transfer a spinal accessory nerve to the suprascapular nerve and/or a branch of the radial nerve innervating the triceps brachii muscle to the axillary nerve for reconstructing their shoulder joints, except for one who had a C5-C8 injury with spinal accessory nerve palsy [6,7].

### Postoperative rehabilitation

In all patients, the operative limb was immobilized in a splint with the elbow at 90° flexion and the shoulder at 0° abduction and flexion and 80-90° inner rotation. Active and passive finger flexion and extension exercises were allowed for all patients just after the surgery. Patients with PUNT were encouraged to apply forces on the muscles innervated by the ulnar nerve three times a day for 20 minutes each from the next day after surgery. Starting from two weeks after surgery, they were allowed to start shoulder motion and elbow flexion and extension exercises of the affected upper extremity with the aid of therapists or family members who had received education from therapists on how to exercise the upper extremity. The upper extremity was kept immobilized in the splint until six weeks after the surgery when the patients were not involved in the rehabilitation program. For those patients with a shoulder subluxation caused by coexisting C5 nerve root injuries, the splint was worn until recovery of the shoulder muscles.

Patients who had undergone ICNT were allowed to apply force to the intercostal muscles in the inspiratory phase of respiration, seven to 10 days after the surgery, after anterior chest pain had reduced. They started passive flexion and extension elbow exercise three weeks after the surgery. However, any shoulder movement was prohibited until five weeks after the surgery.

All patients underwent rehabilitation therapy once a week for three to four months after surgery at our rehabilitation center. After that, they continued to visit the center every two weeks or so to undertake self-performing rehabilitation, and the progress of muscle power recovery around the elbow and shoulder was checked.

### Postoperative assessment

The manual muscle test (MMT) was performed on each patient two or three times a month after surgery and was expressed using Medical Research Council scores [8]. The time required to obtain grades M1 and M3 for elbow flexion and the full range of elbow flexion against gravity with maximum extension of the wrist and fingers after surgery, and the MMT score for elbow flexion at the final examination were investigated on each patient by an investigator blinded to the surgery or preoperative conditions of the patients. Flexion angle of the affected elbow joint more than 110° against gravity was regarded as full flexion of the joint. All patients could extend their elbow joints to 0° actively or with the aid of the gravity before the final examination. Obtaining the full range of elbow motion against gravity with maximum extension of the wrist and fingers meant that patients could flex the elbow joint from 0° to more than 110° against gravity while trying to stretch the finger and wrist, keeping a neutral position as much as possible. Because patients with C5-C8 nerve root injuries cannot extend the wrist or fingers, they were regarded as obtaining the same target when they could flex the elbow joints from 0° to more than 110° against gravity without bending the wrist or fingers of the affected upper limbs. The times to obtain grades M1 and M3 in the above exercises were expressed in postoperative weeks. One week was added to the record when at least four days had passed.

### Statistical analysis

Outcomes in weeks were expressed as the mean and standard deviation, and the ICNT and PUNT groups were compared using nonpaired Student's *t*-tests. The numbers of injured nerve roots and the final MMT scores were compared between the two groups using the Mann-Whitney nonparametric *U *test. Statistical significance was set at *P *< 0.05.

## Results and Discussion

There were no significant differences in the mean age of patients at the time of surgery or in the mean interval between the injury and surgery between the ICNT and PUNT groups. There were significantly more injured nerve roots in the ICNT group than in the PUNT group (*P *= 0.0006) (Tables 3, 4, &5).

**Table 3:** Postoperative Data of the PUNT Group

Pt	M1(W)	M3(W)	M3 with ext f&w (W)	Final MMT
1	3	35	56	4

2	15	43	78	4

3	7	39	72	4

4	12	45	88	4

5	5	24	40	5

6	6	31	52	4

7	24	55	92	3

8	6	22	42	5

Note. The time required to obtain M1, M3 in the elbow flexion, the time required for full flexion of the elbow joint against the gravity with the wrist and fingers maximally extended, and the final MMTs of the elbow flexion. M3 with ext f&w: full flexion of the elbow joint against the gravity with the wrist and fingers maximally extended. MMT: manual muscle test

**Table 4:** Postoperative Data of the ICNT Group

Pt	M1(W)	M3(W)	M3 with ext f&w (W)	Final MMT
9	25	71	83	4

10	31	88	101	4

11	12	52	60	4

12	15	61	78	3

13	21	82	99	3

14	12	48	52	4

15	14	50	58	4

16	13	48	48	4

Note. The time required to obtain M1, M3 in the elbow flexion, the time required for full flexion of the elbow joint against the gravity with the wrist and fingers maximally extended, and the final MMTs of the elbow flexion. M3 with ext f&w: full flexion of the elbow joint against the gravity with the wrist and fingers maximally extended. MMT: manual muscle test

**Table 5:** Average Values and Statistics

	PUNT	ICNT	p value
Age of Surgery	37.9	38.3	0.95

Number of NRs	2.1	3.6	*0.0006

Injury-Surgery	20.3	19.5	0.42

F/U	140.6	130.6	0.83

M1	9.8	17.9	*0.04

M3	36.8	62.5	*0.002

M3 with ext f&w	65.0	72.4	0.42

Final MMT	4.1	3.8	0.20

M3 with ext f&w: full flexion of the elbow joint against the gravity with the wrist and fingers maximally extended, NRs: injured nerve roots, F/U: follow-up period, *: Statistical significance

Two of eight patients in the ICNT group needed drainage of the thoracic cavity because of a postoperative pneumothorax. Five of eight patients in the PUNT group complained of abnormal sensations (hypesthesia and paresthesia) in the ulnar nerve area in the hand; however, this disappeared within 11 days after surgery (mean 7.8 days). No patients showed apparent motor deficits in muscles innervated by the ulnar nerve after surgery. The mean times to obtain M1 in the PUNT and ICNT groups were 9.8 and 17.9 weeks, respectively. The PUNT group required significantly less time to obtain M1 in the elbow flexion than the ICNT group (*P *= 0.04). The PUNT and ICNT groups obtained M3 on average 36.8 and 62.5 weeks, respectively, and the PUNT group obtained M3 significantly faster than the ICNT group (*P *= 0.002). The mean times to obtain the full range of elbow motion against gravity with maximally extended wrist and fingers in the PUNT and ICNT groups were 65.0 and 72.3 weeks, respectively. This was not statistically significant (*P *= 0.42). The mean final MMT score of the PUNT group was greater than that of the ICNT group; however, no significant difference was found between the groups (*P *= 0.20) (Tables 3, 4, &5).

The times required to obtain M1 and M3 in the elbow flexion was significantly shorter in the PUNT group than in the ICNT group. However, there was no significant difference between the two groups in the time required to obtain full flexion of the elbow joint with the fingers and wrist extended. Patients in the PUNT group showed restoration of elbow flexion function more quickly but took more time to separate the finger and wrist motion from the elbow flexion than did the patients in the ICNT group.

In 1994, Oberlin et al. performed PUNT to the MCN on patients with upper BPIs and reported excellent recovery of elbow flexion without noticeable neural deficits in the ulnar nerve [1]. Nowadays, Oberlin's operative procedures are becoming the gold standard to restore the elbow flexion function in patients with C5 and C6 nerve root injuries [2].

There are three reasons for the successful outcomes of PUNT applied to a branch of the MCN innervating the BBM. One is the close proximity of the stump of part of the ulnar nerve and the BBM branch of the MCN, because a part of the ulnar nerve is harvested at the level from which a branch innervating the BBM arises from the MCN trunk [1,2]. Because the neurorrhaphy between the ICN and MCN was performed at the level of the upper margin of the axillary skin fold, the distance between the site of neurorrhaphy (between the ICNs and a branch of the MCN innervating the BBM) and the neuromuscular junction of the BBM was more than 5-6 cm in the ICNT procedure. This is probably why the recovery of normal elbow flexion was faster in the PUNT group than in the ICNT group.

Another reason for the success of PUNT is that the stump of the PUN can be approximated directly to a branch innervating the BBM in the PUNT procedure [1,2]. All nerve fibers regenerating from a stump of the PUN can extend to the BBM and innervate the muscle. Intraneural dissection of the MCN can separate the motor segment (a branch innervating the BBM) from the sensory segment (mainly the lateral cutaneous forearm nerve). The ICN bifurcates into a motor branch (main trunk) and a sensory branch (the lateral branch). We connected the motor branches of the ICNs to the motor segment of the MCN, and the sensory branches (lateral branches) of the ICNs to the sensory segment of the MCN, to allow motor axons of the ICNs to extend selectively to the BBM. Our ICNT could prevent the motor-sensory misdirection of axons extending from the ICNs to the MCN. This might be why there was no significant difference in the final MMT scores for elbow flexion between the PUNT and ICNT groups.

The final reason for the success of PUNT is that the PUN stump contains many motor nerve fibers. The ulnar nerve contains many motor axons because it innervates finger and wrist flexors in the forearm and most intrinsic muscles in the hand but serves for sensation over a relatively small area. Moreover, the ulnar nerve is not functionally distributed, and the motor and sensory axons are still mixed at the level at which the BBM branch arises from the MCN trunk [1]. Thus, a part of the ulnar nerve harvested at the level of the MMB branch of the MCN also includes many motor axons [1,2]. In contrast, the motor branch (main trunk) of the ICN includes not only motor axons innervating the intercostal muscles but also axons serving sensation in the mid-chest. This might explain why the PUNT group demonstrated stronger flexion power of the BBM with minimum motor and sensory morbidity in the ulnar nerve area, although no significant difference was found statistically.

Patients with PUNT bend their affected elbow joints using the ulnar nerve, while those with ICNT do this using the ICN. The ulnar nerve innervates muscles controlling motion of the fingers and wrist. On the other hand, the ICNs are not related to manual function but to respiration. This might be why patients subjected to PUNT spent more time in separating the elbow flexion from the finger and wrist motion than did those following ICNT. Studies on brain plasticity using magnetic resonance imaging or electrophysiological stimulation have revealed several interesting relationships between peripheral nerves and the central nervous system. Thus, the primary motor cortex administrating the intercostal muscles was activated when patients subjected to ICNT tried to flex their elbow joints just after they obtained elbow flexion function. However, the activated motor area moved back to the original area of elbow flexion as elbow flexion power increased [9-11]. Given the results of the present study, we suspect that it would take longer to move the activated cortical area back to the original area controlling elbow flexion in patients subjected to PUNT rather than ICNT. Actually, on the motor homunculus (a functional map of the cortical motor area), the distance between the hand and the elbow is much longer than that between the thorax and the elbow [12].

Some authors have recommended transferring three ICNs to the MCN for elbow flexion. In our experience, the size of two motor branches (main trunks) of the ICNs matches that of the motor segment of the MCN (innervating the BBM), and two sensory branches (lateral branches) of the ICNs also match the sensory segment of the MCN (innervating mainly the lateral forearm skin) in size when the neurorrhaphy is performed between the ICNs and MCN at the level of the upper margin of the axillary skin fold. According to a meta-analysis of the elbow flexion function using ICNT, the authors concluded that there was no statistically significant difference in elbow flexion between double or triple branch transfer of the ICNs to the MCN [13].

We transferred the sensory branches of the ICNs to the sensory segments of the MCNs, even in patients with C5-C8 nerve root injuries in the ICTS group. All patients of this group recognized sensation in the lateral forearm area at the final follow-up, probably because most axons regenerated from the sensory branches of the ICNs had extended the lateral forearm cutaneous nerve (the terminal branch of the MCN). It might have been an option to transfer the sensory branches of the ICNs to the median nerve with the lateral cord origin to restore the sensation of the hand in patients with C5-C8 injuries. Because of the transfer of a small number of the axons (only two sensory branches of the ICNs), a long distance between the site of the neurorrhaphy and the target tissue (hand) and occurrence of the axon misdirection in the median nerve, functional sensory recovery in the hands was not expected in these patients.

Recently, some investigators have harvested motor dominant funiculi of the ulnar nerve, which innervate the wrist or finger flexor muscles rather than the intrinsic muscles of the hand, during PUNT surgery in order to restore strong elbow flexion and avoid occurrence of significant motor deficits of the ulnar nerve [2,14,15]. Funicular dissection and electric stimulation is necessary to identify motor dominant funiculi that mainly innervate the wrist or finger flexors. Funicular dissection is associated with a risk of injuring healthy nerves. Oberlin et al. mentioned that each funiculus of the ulnar nerve was not functionally distributed at the level of the upper arm where the BBM branch was separated from the MCN and that harvesting a funiculus did not cause any significant deficits in ulnar nerve function [1]. We were concerned about the occurrence of neurogenic pain after the funicular dissection of the ulnar nerve after the PUNT. Thus, we harvested the lateral funiculi of the ulnar nerve (they are the closest to the BBM branch of the MCN) with great care and minimum invasiveness to the nerve, which may have prevented the occurrence of donor nerve problems after the PUNT.

The weakness of the present study was the significant difference in the number of injured nerve roots between the two groups. In the PUNT group, all patients had injuries to the C5 and C6 nerve roots except for one patient with a C5-C7 injury. In the ICNT group, three had C5-C7 injuries and five had C5-C8 injuries. Ideally, the study should be performed on patients with the same type of brachial plexus injuries. Possibly, elbow flexion might have been facilitated by Steindler's effect [16,17] in patients in the PUNT group, because the finger and wrist flexors of the patients were functioning.

## Conclusions

The PUNT procedure is technically easy, not associated with significant complications and provides rapid recovery of elbow flexion. However, the time needed to separate elbow flexion from finger and wrist motion was significantly longer with PUNT than with ICNT. Although the mean final MMT score for elbow flexion in the PUNT group was greater than that for the ICNT group, no statistically significant difference was found between groups for this factor.

## Abbreviations

PUNT: Partial ulnar transfer; BPI: Brachial plexus injury; ICNT: Intercostal nerve transfer; MMT: Manual muscle test; BBM: Biceps brachii muscle; MCN: Musculocutaneous nerve; PUN: Partial ulnar nerve; ICN: Intercostal nerve.

## Competing interests

The authors declare that they have no competing interests.

## Authors' contributions

RK performed surgery on all patients and owed the final responsibility in the present study. RI, KN and TM assisted RK in the operations. SO was not involved in any operations and performed only the postoperative assessment of each patient. SD was blind to the patients and analyzed the preoperative and postoperative data to draw the conclusions. TK performed personal and financial management in the study. All authors read and approved the final manuscript.
